# An assessment of health research impact in Iran

**DOI:** 10.1186/s12961-016-0129-9

**Published:** 2016-07-26

**Authors:** Bahareh Yazdizadeh, Reza Majdzadeh, Leila Janani, Farideh Mohtasham, Sima Nikooee, Abdmohammad Mousavi, Farid Najafi, Maryam Atabakzadeh, Azam Bazrafshan, Morteza Zare, Manoochehr Karami

**Affiliations:** 1 Knowledge Utilization Research Center, Tehran University of Medical Sciences, Tehran, Iran; 2 Epidemiology and Biostatistics Department, Tehran University of Medical Sciences, Tehran, Iran; 3 Department of Biostatistics, School of Public Health, Iran University of Medical Sciences, Tehran, Iran; 4 Social Determinants of Health Research Center, Yasuj University of Medical Sciences, Yasuj, Iran; 5 Research Center for Environmental Determinants of Health, Kermanshah University of Medical Sciences, Kermanshah, Iran; 6 Bushehr University of Medical Sciences, Bushehr, Iran; 7 Neuroscience Research Center, Institute of Neuropharmacology, Kerman University of Medical Sciences, Kerman, Iran; 8 Social Determinants of Health Research Center and Department of Epidemiology, School of Public Health, Hamadan University of Medical Sciences, Hamadan, Iran

**Keywords:** Research impact assessment, Payback, Health research system

## Abstract

**Background:**

In recent years, Iran has made significant developments in the field of health sciences. However, the question is whether this considerable increase has affected public health. The research budget has always been negligible and unsustainable in developing countries. Hence, using the Payback Framework, we conducted this study to evaluate the impact of health research in Iran.

**Methods:**

By using a cross-sectional method and two-stage stratified cluster sampling, the projects were randomly selected from six medical universities. A questionnaire was designed according to the Payback Framework and completed by the principle investigators of the randomly selected projects.

**Results:**

The response rate was 70.4%. Ten point twenty-four percent (10.24%) of the studies had been ordered by a knowledge user organization. The average number of articles published in journals per project was 0.96, and half of the studies had no articles published in Scopus. The results of 12% of the studies had been used in systematic review articles and the same proportion had been utilized in clinical or public health guidelines. The results of 5.3% of the studies had been implemented in the Health Ministry’s policymaking. 62% of the studies were expected to affect health directly, 38% of them had been implemented, and among the latter 60% had achieved the expected results. Concerning the economic impacts, the most common expected impact was the reduction of ‘days of work missed because of illness or disability’ and impact on personal and health system costs. About 36% of these studies had been implemented, and 61% had achieved the expected impact.

**Conclusion:**

In most aspects, the status of research impact needs improvement. A comparison of Iran’s ranking of knowledge creation and knowledge impact in the Global Innovation Index confirms these findings. The most important problems identified were, not conducting research based on national needs, and the lack of implementation of research results.

**Electronic supplementary material:**

The online version of this article (doi:10.1186/s12961-016-0129-9) contains supplementary material, which is available to authorized users.

## Background

Where scientific development is concerned, Iran has experienced the fastest rate of growth among countries in the Middle Eastern region in the past 30 years [1]. In the field of health sciences it has also seen a significant development, such that Iran’s scientific health articles published during the years 1990–2009 was 36.5 times the number published during 1980–1989 [1]. The important question that arises is whether this considerable increase in scientific production in health sciences has affected public health and welfare. Have we tried to implement the results as much as we have tried to produce them? Furthermore, the research budget has always been negligible and unsustainable in developing countries. Nevertheless, in Iran, the research budget has increased from 0.55% of the Gross Domestic Product (GDP) in 2001 to 0.87% in 2009, and was meant to be raised to 2.5% in 2015, although this did not happen. It appears that the real reason behind the failure to secure a research budget and its lack of sustenance, however, is the policymakers’ lack of belief in the positive impacts of research compared to other investments [2]. Measuring the impact of research and identifying the points requiring intervention are the first steps toward improving research impact. Moreover, by investigating the impacts of health research we may achieve basic data for future assessments of interventions.

Few studies have been conducted to examine the impact of health research. The impact and benefits of health research can be measured in two ways, namely through top down (ecologic studies) or bottom up (case studies) approaches [3]. In top down studies, variables used at the global level are collected such as an assessment of the association between the cost of research and its impact on the reduction of mortality and morbidity resulting from specific diseases [4–6]. In bottom up studies, a number of projects are assessed for their impacts. One of the most important models recommended for assessing the impact of health research through the bottom up approach is the ‘Payback Framework’, presented by the Health Economic Research Group of Brunel University London in the United Kingdom, to investigate the impact of health research [7]. In this framework, the resultant benefits of health research are classified into five categories of ‘knowledge advancement’, ‘capacity building’, ‘impact on decision-making’, ‘health impact’, and ‘social and economic impact’ [7–10]. In 2009, the Canadian Academy of Health Sciences introduced detailed indicators for each of the domains defined in the Payback Framework [11]. A small number of studies have been conducted in developed countries to assess the impact of research through this framework and its proposed indicators [12–15], all of which approve its appropriateness. However, the impact of research in developing countries has not yet been assessed through this framework [16].

In order to judge the impact of research, three successive questions must be answered: is the research performed tailored to local needs? Are the research results implemented? And, are the desired results achieved? The present study was designed and conducted using the Payback Framework to address the applicability of these three questions in Iran.

## Methods

### Target population and sampling

Based on university size and capacity, Iran’s universities of medical sciences are classified into three categories. Here, we applied a two-stage stratified cluster sampling to stratify the universities according to the three categories and we selected two from each stratum. Prior to 2007, universities were not required to register their project data in electronic databases, and therefore data extraction prior to that date was not possible. The present study includes the list of projects completed by 2007 and 2008 by the selected universities. The projects were randomly selected from each university, with 200 projects being selected from type 1 universities and 100 projects from the other two categories. Since the number of completed projects in type 3 universities was very low, these were all included in the study.

Based on recommendations made by previous studies, in order to provide an accurate picture of the impact of research, sampling must be purposeful and based on variables affecting impact, such as the type of study and the activities undertaken to disseminate and implement the research results, or not [17]. However, the documentation available from the universities’ Deputies of Research had not classified the studies based on their types, and therefore we were unable to conduct sampling based on these grounds. Further, due to the lack of available data, sampling based on the activities undertaken to disseminate results or the impact research was not possible. Moreover, performing a survey on all the research performed at all six universities was not possible in terms of human power and costs. Therefore, under the existing circumstances, random sampling was the best choice.

Each academic member (principle investigator of each project) completed the questionnaire for the selected project; if someone had many completed projects, only one of their projects was included in the study.

### Design and standardization of the questionnaire

A questionnaire was developed using the indicators presented in the ‘Making an Impact’ report [11] and studies conducted in Hong Kong [12] and Australia [13] to measure the impact of health research based on the Payback Framework.

The questionnaire provided to the researchers first explained the objective of the study and then asked researchers to specify their type of research on the basis of the following three classifications:

- ▪ The classification proposed by the Organization for Economic Co-operation & Development (OECD) [17];
- ▪ The classification proposed by the Australian National Health and Medical Research Council [18];
- ▪ The classification based on the methods of the study.

Then, questions were asked for each impact domain, as indicated below.

#### Knowledge advancement

Researchers were asked to indicate the articles published in journals and presented at conferences (the articles specified in the questionnaire were searched for in Scopus and their citation rates were retrieved).

#### Capacity building

Researchers were asked whether the research project was a thesis and the number of students involved in it; whether the research team had acquired new skills; whether there had been empowerment of the study’s target group (participatory research); the degree of strengthening of organizational research resources; the channels through which all or some of the infrastructures required by the project were achieved; whether research budgets for future projects had been facilitated from the same or other organizations; and whether the project results had been utilised by the researcher (of the same project) and/or other researchers to define further projects.

#### Impact on decision-making

The researchers were asked about the utilization of research results in systematic reviews relevant to decision making; the utilization of research results in documentations relevant to decision making, including clinical guidelines or public health guidelines, health technology assessment (HTA), educational content for patients and/or the public, policy briefs, policy bills, guidelines, and/or legislations of executive organizations; the utilization of research results in book compilation and development of educational content for professional groups (students’ continuing education and/or training); the utilization of research results in policymaking in the local context, directly or indirectly, inside and outside the Ministry of Health and Medical Education (MOHME); the registration of domestic or international patents; and whether the aforementioned research was conducted upon the demand of a specific organization and/or policymakers and managers and/or the industry.

#### Health impact

The researchers were asked to define the expected impact of their research results on the level of health (such as the prevalence and incidence of disease, quality of life and/or life expectancy), on the status of health determinants (such as modifiable risk factors, social determinants and bio-environmental determinants), and on the quality of health service provision (acceptability, accessibility, appropriateness, effectiveness, efficacy and safety) as well as the extent of implementation of results, achievement of the desired result in practice, and the share of research results in the changes created.

#### Economic impact

The researchers were asked about the expected impact of research results with regards to a direct effect on the production of materials or consumer services, optimizing the earlier goods and/or products (increasing quality and/or reducing production costs), creating knowledge-based entrepreneurship, decreasing days of work missed because of illness or disability, and the impact on direct patient costs and the health system, as well as on the extent of implementation of results, achievement of the desired result in practice, and the share of research results in the changes created.

The questionnaire is available in Additional file 1.

#### Questionnaire validity

We used expert opinion to investigate the face and content validity and to identify other domains not considered in the Payback Framework and indicators. To this end, we invited academic experts from various disciplines to participate in the panel. Sixteen experts from the following specialties participated in the panel: drug management and economy, neurology, neurosurgery, nutritional sciences, endocrinology and metabolism, cardiovascular diseases, psychiatry, traditional medicine, pharmacology, sports medicine, community medicine, health services management, and general physicians. We then asked the participants to complete the questionnaire for one of their projects that had been completed 3 years prior. Then, all the sections and questions were discussed in succession. The discussions were audio-recorded and transcribed upon permission from the participants. The research team examined the changes requested in the panel, and implemented them in the questionnaire upon approval.

#### Questionnaire reliability

We used the test–retest method for this purpose. In the first round, the questionnaire was sent to 14 individuals. After a month, the questionnaire was sent to them again. The reliability of the questions was measured with Kappa. Nine questions had kappa < 0.6; by changing the structure of the questions and their wordings, we tried to raise their reliabilities. Furthermore, if their research results had been implemented in decision-making and/or resulted in health and economic impacts, we asked researchers to provide documentation to this effect. At this stage, we realized that bringing forth such documentation was difficult for the researchers and therefore they preferred to avoid choosing the ‘yes’ option. Thus, we re-considered our request and asked them only to name the documentation. Then, taking into account the responses given and the type of study (methodological classification), the research team decided about the validity of the responses.

To examine the number of article citations, the titles that had been outlined by the researchers in the questionnaire were searched in Scopus from January to May 2015, and their citations were registered.

### Analysis

Double entry of data (of the completed questionnaires) was performed. Eventually, the two databanks were compared with Epi Info software, the inconsistent cases were examined, and the final file was produced.

Considering that stratified cluster sampling was performed, the stratification and cluster effects were considered in the analysis. We performed data analysis with Stata 13 and survey (svy) commands. Standard error calculation was not possible for some of the variables due to strata with single sampling units. Under these conditions, standard error was calculated by removing the effect of weighting.

Regarding the section related to the health and economic impacts, since some of the questions were correlated content-wise, whereas some were combined with each other, so that the health and economic impacts were presented with one and five indicators, respectively.

The study has been approved by the Institutional Review Board of Tehran University of Medical Sciences which abides by the Helsinki Declaration.

## Results

Overall, of the 402 research projects, 283 project executives completed the questionnaires (70.4% response rate). According to the researchers, 10.24% (SE = 0.02) of the studies had been ordered by a specific organization and/or policymaker, manager and/or the industry. The results have been presented in separate tables for each of the Payback Framework’s domains (Tables 1, 2, 3, 4 and 5).

**Table 1:** Knowledge advancement in the projects completed by 2006 and 2007

Impact	Relative frequency estimate, % (standard error)
Projects that have produced no articles	33 (0.04)
Projects that have at least one article published in Scopus	50 (0.09)
Impact	Mean (standard error)
The average number of citations made to the article in Scopus	11.3 (1.8)^a^
	(The median of this index is 5.5)
The average number of articles published in journals per project	0.96 (0.14)
The average number of articles presented at conferences per project	0.13 (0.03)

^a^Standard error was calculated by removing the effect of weighting

**Table 2:** Estimates of relative frequencies of projects completed by 2006 and 2007 for different classifications, respectively

The classification proposed by Europe’s OECD							
Experimental developmental			Applied			Basic	
10.8% (0.03)			80.2% (0.04)			9% (0.01)	
The classification proposed by the Australian national health and medical research council							
Public health		Clinical research		Health services research		Strategic basic research	Pure basic research
9% (0.01)		29.3% (0.02)		11.5% (0.02)		44.1% (0.03)	6% (0.01)
Classification based on method of study							
Descriptive observational	Analytical observational	Interventional	Systematic review	Diagnostic value of tests	Laboratory studies	Design and production of equipment, software, medicines or chemical substances	Others
22.5% (0.03)	34.8% (0.02)	15.0% (0.02)	1% (0.01)	2.4% (0.01)	15.6% (0.02)	4.3% (0.02)	4.7% (0.02)

**Table 3:** Capacity building by the projects completed by 2006 and 2007

Impact	Relative frequency percent, % (standard error)
Thesis for one degree	47.3 (0.03)
Thesis for two degrees	1.5 (0.009)
The average number of students per project	0.6 (0.07)
Acquisition of new skills in the research group	77.3 (0.03)
Empowerment of the target group	32.5 (0.03)
Strengthening the organization’s research resources	30.6 (0.03)
Preparing part or all of the required infrastructures of the project through another channel	34 (0.05)
Facilitating the securing of research budget from other organizations	13.9 (0.01)
Utilization of project results by the researcher and/or other researchers to define the following projects	47.7 (0.06)

**Table 4:** Impact on decision making by the projects completed by 2006 and 2007

Questions	Percentage of relevant projects, % (standard error)	Percentage of relevant projects that have been utilized, %^a^ (standard error)
Utilization in systematic reviews	NA	12 (0.04)
Utilization in clinical guideline or public health guideline	71 (0.03)	11.7 (0.03)
Utilization in health technology assessment	34.0 (0.03)	1.6 (0.008)
Utilization in educational content for patient and/or the public	34.4 (0.02)	9.8 (0.02)
Utilization in policy briefs	27.5 (0.02)	2.0 (0.01)
Utilization in policy bills, guidelines and/or legislations of executive organizations	47.2 (0.04)	16.3 (0.03)
Utilization in book compilation	96.6 (0.009)	8.2 (0.03)
Utilization in development of educational content for professional groups (education or continuing education of academic students)	93.2 (0.02)	3.4 (0.02)
Utilization in policymaking by the Health Ministry (directly or indirectly)	85.2 (0.02)	5.3 (0.01)
Utilization in policymaking outside the Health Ministry (directly or indirectly)	81 (0.02)	0.9 (0.006)
Utilization in decision making in local contexts	87.7 (0.02)	29.0 (0.006)
Registration of domestic patents	77.1 (0.02)	1.5 (0.008)
Registration of inventions or foreign patents	76 (0.02)	0.5 (0.003)

^a^All the percentages in the column are dependent on the previous column

NA: The researchers believe that any type of project can be relevant in terms of being utilized in systematic reviews

**Table 5:** Health and economic impact of the projects completed by 2006 and 2007

	Relative frequency of projects with the expected impact, % (standard error)	Relative frequency of projects whose results have been applied, % (standard error)^a^	Relative frequency of projects which have achieved the expected impact, % (standard error)^a^		
Health impact				The share of research in the final change, %	Relative frequency of the share of research in the final change, %^b^
Direct impact on health, health determinants, or quality of delivered services	62 (0.04)	38 (0.05)	59.7 (0.04)	<25	45.9 (0.09)
				25–50	23.32 (0.08)
				50–75	5.42 (0.05)
				>75	25.36 (0.07)
Economic impact					
Production of new products or improvement of already existing products	9.5 (0.03)	27.7 (0.08)^b^	55 (0.20)^b^	<25	5 (0.29)
				25–50	0
				50–75	25 (0.25)
				>75	25 (0.25)
Knowledge-based entrepreneurship	8.5 (0.02)	9.4 (0.07)^b^	100 (0.00)^b^	<25	100 (0.00)
				25–50	–
				50–75	–
				>75	–
Reduction of days of work missed because of illness or disability	36.9 (0.06)	36.6 (0.05)	60.8 (0.09)^b^	<25	47.71 (0.14)
				25–50	22.55 (0.11)
				50–75	10.16 (0.07)
				>75	19.57 (0.11)
Reduction of patient direct costs	42.5 (0.02)	37.8 (0.04)	63.9 (0.08)^b^	<25	22.37 (0.09)
				25–5	30.37 (0.10)
				50–75	24.88 (0.09)
				>75	22.37 (0.09)
Reduction of health systems costs	45.3 (0.04)	32.6 (0.03)	59.8 (0.08)^b^	<25	22.44 (0.11)
				25–50	12 (0.07)
				50–75	45.14 (0.12)
				>75	20.41 (0.09)

^a^All the percentages in the column are dependent on the previous column

^b^Standard error was calculated by removing the effect of weighting

## Discussion

The current study was conducted to investigate the impact of health sciences research in Iran through the bottom up approach by the Payback Framework.

To judge the appropriateness of impact, we need an acceptable standard for each of the questions and domains, which does not currently exist, and is itself one of the challenges of assessing research impact [19]. Appraising some of the questions is not easy; nevertheless, the least benefit of these data is that they can be used as basic data for future assessments.

The strengths of the study include the large number of projects from various universities, and the acceptable response rate, which strengthens the trust in the results obtained.

Nevertheless, one of its most important limitations is the study method involving using researchers’ opinions [20], the major presumption of which is that their responses are valid. Although this presumption is largely acceptable, considering that the content of the questions was new to the researchers, certain measures had to be taken to raise the validity of the responses. Another weakness of the study is the unavailability of project costs, which is a significant variable in performing knowledge translation activities and for research impact. Although project costs had been included in the questionnaire, the number of missing replies was too high and invalid, so we were unable to analyse the given responses.

### Knowledge advancement

The average number of articles produced from each project was different from what was observed in previous studies; 0.96 versus 2.3 in ‘primary research impact’ in Australia [13] and 5.4 in ‘health service research impact’ in Hong Kong [12]. Although it would have been more realistic to judge the appropriateness of this number had we had access to project costs, considering that a third of the studies had not produced any publications and that more than half of publications had not been included in Scopus, we may deduce that article production from research projects as high as would be desired. Moreover, the number of articles presented in conferences is also small, which indicates the little interaction among researchers. Half of the articles produced had been included in Scopus, but had been cited fewer than 5.5 times. Thus, the quality of the knowledge produced requires particular attention.

The distribution of studies does not match the country’s needs; only 10% were of the experimental developmental type. Considering the country’s need for such studies, where the goal is to implement research-derived information or practical experience to produce materials, novel products or devices, innovative procedures, systems or new services, and/or major improvement of already existing products [17], this figure is small. Furthermore, almost 44% of the studies were basic science studies with specific applied goals. As Wooding et al. [15] stated in their study examining the impact of cardiovascular research, basic science studies that have a defined clinical application are more influential than pure basic science studies. Only about 20% of studies were health services research and/or public health research. These studies usually address local issues. Some research results are applicable in all settings, e.g. research on the efficacy of drugs. However, some issues, such as the burden of disease or the best way to deliver effective interventions, need local answers. Therefore, every country needs to produce local evidence for its local health issues [16]. In light of the limited resources available in developing countries, the need to conduct these types of studies is greater. Only 1% of the studies were systematic reviews, while these studies have a special place in policymaking [21–23].

### Capacity building

Half the studies were theses, which culminate in educating and training researchers. Iran ranks fourth in the Global Innovation Index 2015 report on tertiary education [24]; hence, its growth in the higher education arena seems to have played a significant role in its scientific development. The average number of students per research project in the current study is similar to the number reported in Britain’s ‘asthma research impact’ study by Hanney et al. [9].

Furthermore, over two-thirds of the projects have resulted in the acquisition of new skills by the research team. About one-third of the studies conducted resulted in empowering the target group. However, considering that a small number of projects have been conducted with community participation or another stakeholder, this ratio is not surprising. Approximately a third of the studies have led to the strengthening of the research organization’s resources. However, the accuracy of this proportion is difficult to ascertain.

Overall, 13.9% of the research projects facilitated the securing of research grants from other organizations, which indicates the need for that research and the trust the organizations have in the researchers. Therefore, one may hope that the results of these 13.9% research studies will be implemented. Moreover, a specific organization, policymaker, manager, and/or the industry have ordered 10.24% of the studies. Other studies in Iran have estimated this figure at 11.8% [25] and 16% [26]. This figure is similar to that of securing research grants from other organizations.

Almost half the project results had been used by the researcher (themselves) or other researchers to define future projects. This rate was 32% in another study conducted in Iran [26], whereas in studies conducted in other countries it was reported at 23%, 44.9% and 65% [9, 12, 13]. This shows that half of the studies are not related to their subsequent studies, a point worth reflecting upon. The reason behind this matter must be sought in the researchers’ incentives toward research topic selection.

### Impact on decision-making

A total of 12% of the study results had been utilized in systematic review studies. This figure is strikingly different from the 100% rate reported by Reed et al. [13] in Australia. The reasons behind the lack of utilization of research results in systematic reviews include the lack of availability of these studies and their poor quality. Nevertheless, since half the studies have not published their articles in Scopus, this number is not surprising.

Regarding the utilization of research results in documentations required for decision-making, the most common application was in policy bills, guidelines and/or legislations of executive organizations. The least number of cases of application were in HTA and policy briefs, which, considering the small number of HTA and policy briefs produced in the country, is not beyond expectation. Nevertheless, the fact that, in spite of their relevancy, only 16.35% of studies have been applied in policy documents is a major problem. A study investigating the impact of primary research in Australia reported the rate of information development for policymaking at 77% [13], which is considerably different from our results. The above matter also holds true for book compilation and educational content development, as these materials can also influence decision-makers.

The rate of research result application in policymaking (directly or indirectly) varied from 0.93% (outside MOHME) to 29% (local policymaking). Elsewhere, this rate varies from 13% in Britain’s ‘asthma research impact’ study [9] to 31% in the study on ‘primary research impact’ in Australia [13]. Therefore, the figures observed here greatly differ from those in other countries.

### Health and economic impact

A little over half of the studies could have directly influenced health; however, only a third of them have been implemented. Nevertheless, in cases where they have been implemented, half have achieved the desired result, which indicates that half have not influenced health. In a country with limited resources for research, this figure may seem high, but the worrisome issue is the minor implementation of results, which necessitates serious interventions. Overall, 14% of studies have resulted in changes in health outcomes in the present study, which varies from the 10% reported by the British ‘asthma research impact’ study [9] and the 50% by the ‘primary research impact’ Australian study [13].

We observed the same pattern with regards to economic impact. The interesting point of this section is that the number of studies that were expected to result in knowledge-based entrepreneurship was small. However, had the results of these studies been implemented, they would have culminated in the desired result. Overall, 9.94% of researchers stated that their research results had the capability of producing a new product or improving an existing one. Yet, upon comparing this figure with the number of registered patents, we observed that although 10% of the studies could have registered patents, fewer than 2% had resulted in patent registration. This too, indicates the lack of implementation of research results in their potential pathway. Here, we observed that 1.44% of studies had resulted in the manufacturing of a new product or optimization of an already existing one, which, if compared to the 17% reported in the British ‘asthma research impact’ study [9], is a very small figure.

The share of research in the changes brought about was not evenly distributed, although, overall, most projects were believed to have created approximately 25% of the changes. However, the share of influential projects that had affected the direct costs of patients and the health system, and those that had culminated in the manufacture of a product or optimizing one was greater than 25%.

This result was not unexpected either; generally, we do not expect different types of studies to have the same impact. Nevertheless, care must be taken in interpreting these data, as, in addition to the type of study, knowledge exchange activities and contextual factors are other important influential factors affecting research, which we have not investigated in this study.

The comparison of our results with those of other studies is not easy, as the various studies have been conducted with different sample sizes and questions. Moreover, the impact of research is dependent on the context and infrastructure of each country. Hence, the results of different studies from different settings are not directly comparable. We have stated the descriptive characteristics of similar studies in Table 6. In general, there is no standard for the ideal level of research impact. Nevertheless, upon comparing our results with those of other studies extracted, we observed that, in all its aspects, the research impact was smaller in the present study.

**Table 6:** Summarized results of studies that have examined the impact of health research

Variables examined	Publicly funded health and health services research Hong Kong, Kwan et al., 2007 [12]	Primary health care research, Australia, Reed et al., 2011 [13]	Asthma research UK, Hanney et al., 2013 [9]	Cardiovascular research, UK, Canada, Australia, Wooding et al., 2014 [15]
The organization examined	Health and health research fund	All primary health care research studies funded by the National Health Medical Research Council, the Aboriginal Health Medical Research Council, the General Practice Evaluation Program, the Cooperative Research Council for Aboriginal Health, and the Primary Health Care Research, Evaluation and Development	Charity funding	English department of health
				Canadian Institutes of Health Research
				Charities: The Heart Foundation (Australia), Heart and Stroke Foundation of Canada, British Heart Foundation, UK Stroke Association
Type of studies	Public health and health services research	Interventional descriptive	Not specified	Basic clinical
Number of projects	205	41	153	29
Response rate	86.8%	49%	59%	100%
Results: knowledge advancement	5.4 articles per project 295 projects have been cited in ISI and social sciences, wherein each article has been cited 1.9 times on average	2.3 articles per project	4 articles per project	All the studies had academic impact (basic science studies had greater impact than clinical ones); however, the wider impact of clinical studies was greater than that of basic science studies
Results: capacity building	44.9% used for future research	94% used for staff development	45 PhD graduates, 21 MDs	
		65% used for future research	23% used for future research	
Results: impact on decision-making	35.4% impact on informing policy (treatment guideline and protocol, reference standard, Cochrane review)	100% have been utilized in guidelines and systematic reviews	13% of projects have influenced policies	
		77% provided information for policymaking		
	49.4% created change in behaviour or clinical practice in managers, service providers and the public	31% influenced policymaking		
		85% provided information for organizations and 73% influenced it/them		
Results: health and economic impacts	42.1% caused health services benefit (adoption of cost effective strategies, qualitative improvement, improved effectiveness of public health policies, selling of intellectual property rights)	70% improved service delivery	10% have influenced health	
		58% used in clinical practice	17% of projects have resulted in manufacture of products	
		50% improved health outcome	For every pound invested, 1.40 pounds were secured for the next projects from sources other than Asthma UK	

Based on our results, the most important problems identified are not conducting research based on national needs and the lack of implementation of research results. The lack of a systematic process for determining and disseminating health research priorities was introduced as a barrier in another study in Iran [27]. As mentioned by Meagher et al. “*it is impractical to attempt to measure something which one has not deliberately tried to bring about*” [28]. Therefore, if we want to improve the impact of research on decision making (academic or non-academic), we should promote the implementation of research results first. The barriers to implementation of research findings in health decision-making in developing countries have been investigated in a couple of studies [27, 29–31]. Some of these barriers are related to the knowledge generating organizations, and the others are related to knowledge user organizations. Others are seen in the interactions between the two groups. To eliminate these barriers, interventions must be aimed at both sectors and at different levels. Where achievement of results upon applying them is concerned, half the studies have attained the desired results, but the other half have not. This section requires further study to identify the factors affecting this field. The gap between knowledge generation and utilization is a grave problem, which is not only present in the health sector, but also exists in other sectors. In the Global Innovation Index 2015, Iran ranked 24th in the knowledge creation sector, whereas it ranked 114th among 141 countries in the knowledge impact sector [24]. These figures clearly point to the know-do gap.

Two types of research impact must be differentiated from each other in research impact assessment. Firstly, the result of each study is used to design a new one and, secondly, if a research finding has direct application in health and economy it should be implemented. The target audiences of the first group are researchers, and those of the second are policymakers and managers, service providers, patients, the community, and the industry. Each of these study groups requires special interventions to increase research uptake.

Another noteworthy point is that we have examined the studies that have been completed by 2007 and 2008. In the years that followed, certain changes were introduced that may have changed the status of Payback indices. Following the revolution and war, the country’s research policies were focused on increasing scientific productions, a goal that was achieved [1]. Thereafter, macro-level policies were aimed at conducting purposeful research, such as priority-setting in the health system and development of ‘Iran’s Health Innovation and Science Development Plan by 2025’. The latter is a subset of the ‘Comprehensive Scientific Map of the Country’ [32], published in 2009. In this regard, certain interventions were undertaken in Iran’s universities of medical sciences to strengthen the application of research results, as follows:

- ▪ Establishment of knowledge translation units
- ▪ Establishment of the ‘Office of Industrial Relations’ in some universities
- ▪ Conducting knowledge translation workshops
- ▪ Allocation of budget to knowledge translation activities
- ▪ Considering promotion scores for academic members for making their research results applicable
- ▪ Considering the application of research results during evaluation of universities of medical sciences and research centres by MOHME
- ▪ Allocation of 2% of the university budget to applied research
- ▪ Holding workshops on evidence-informed policymaking in various sectors of MOHME
- ▪ Promoting the production of systematic reviews and clinical guidelines
- ▪ Establishment of incubators (places wherein research results that can be commercialized are presented to companies that have been formed by graduates and initial entrepreneurship support is provided) [33, 34]

## Conclusion

In most aspects, research impact needs improvement. A comparison of Iran’s ranking of knowledge creation and knowledge impact in the Global Innovation Index confirms these findings. The most important problems identified were not conducting research based on national needs and the lack of implementation of research results.

In the future, in addition to overcoming the barriers that have existed toward research impact assessment, new indices should be considered for evaluating impact and the pathways for achieving it. Among the indices, co-authorship (at individual and organizational level) indicates the impact of research on establishing research networks among researchers, and can also predict the impact of research. Moreover, the identification and measurement of indices that can quantitatively indicate the path of research should be investigated in future studies.

## Additional file

Additional file 1: Study questionnaire. (DOC 68 kb)
