# Supplementary material for: An assessment of health research impact in Iran
Source: Health Res Policy Syst. 2016 Jul 26;14:56. doi: 10.1186/s12961-016-0129-9 (PMC4962356; doi:10.1186/s12961-016-0129-9)
Supplement: Additional file 1: — Study questionnaire. (DOC 68 kb) [file 12961_2016_129_MOESM1_ESM.doc]

**In the name of God**

The **“Health Research Impact Assessment”** Questionnaire

**Measuring impact**

Dear Sir/Madam,

We hereby inform you that the **‘Health Research Impact Assessment’** project is underway in the ‘Knowledge Utilization Research Center’. Using the ‘payback’ framework, the impact of health research is assessed in five domains of ‘knowledge advancement, capacity–building, impact on decision-making, health and economic impacts’. To this end, after specifying a number of medical universities for inclusion in the study, a number of projects completed by 2007 & 2008 were randomly selected. One of your projects titled ‘…’ has been selected. Therefore, as the principle investigator of the project, we would be grateful if you would complete the questionnaire.

If you find any ambiguous points in the questionnaire, please let us know. For further information, you may contact Dr. Yazdizadeh at 88975658.

Thank you

**Section A: Project classification**

**1- Please specify the type of research for each of the classifications below.**

**Classification no. 1** (the classification proposed by the **Organization for Economic Co-operation and Development**)

🗌Basic (experimental work that is done chiefly to gain new information about the origins of phenomena and observable facts, without any special application or use)

🗌 Applied (a study that is conducted to obtain new information and which is aimed towards specific goals)

🗌 Experimental developmental (a systematic work that is done to utilize research – derived information or practical experience to produce materials, novel products or devices, innovative procedures, systems or new services and/or major improvement of already existing products)

**Classification no. 2** (The classification adopted from the Australian national health and medical research council)

🗌 Pure basic research (studies that are conducted with the sole purpose of advancing knowledge)

🗌 Strategic basic research (studies that have had applied goals at the time of study design)

🗌 Health Services Research (an inter-sectoral research that investigates how social factors, financial systems, organizational structures and procedures, health technologies and individual behavior affect the access to, quality, and costs of health services)

🗌 Clinical research (research conducted on patients or in a clinical context)

🗌 Public health studies (research conducted at public/community level)

**Classification no. 3** (based on the objectives and methods)

🗌 Observational – descriptive studies

🗌 Observational – analytical studies

🗌 Interventional studies (such as clinical trials)

🗌 Systematic reviews

🗌Evaluation of diagnostic tests

🗌Laboratory studies

🗌 Design or production of equipment, software, medicines, and or chemical substances

🗌 Others: ……………………………………………………………………….

**Section B: Advanced knowledge**

**2- Please enter the titles and journal names of the articles published from this research in the table below.**

| Row | Article title | Journal name |
| --- | --- | --- |
|  |  |  |
|  |  |  |
|  |  |  |

**Section D: Capacity – building achieved by conducting the research**

**3- Was part or whole of the research a student thesis?**

🗌 Yes : 1. Master’s degree thesis 🗌 4. MPH thesis 🗌

2. MD thesis 🗌 5. PhD thesis 🗌

3. Clinical residency thesis 🗌 6. Clinical fellowship thesis 🗌

🗌 No

**4- How many students have graduated with the aforementioned research? ……………**

**5- Has this research resulted in the acquisition of new skills by the research team?**

🗌 Yes the type of skill acquired ……………………………..

🗌 No

**6- Has this research resulted in the empowerment of the target group of the study? (One example of such research is participatory research, which is done by voluntary participation of the target group, including the public and/or specific professional groups. Regardless of the research result, these studies can result in capacity – building in the individuals who have participated in the research)**

🗌 Yes The type of capacity – building done ………………………………………………….

The people who have had their capacities built ………………………………….

🗌 No

**7- Has part or whole of the research expenditure been spent on strengthening the research resources of the organization in which you work (such as software or equipment)?**

🗌 Yes 🗌 No

**8- If your response to question no. 9 is yes, what percent of the research budget was spent on strengthening resources?**

🗌Less than 10% 🗌 10 – 25% 🗌 26 – 50% 🗌 More than 50%

**9- Have part or whole of the infrastructures required for this research been acquired through channels other than the own budget of the study?**

🗌 Yes 🗌 No

**10- Has this research facilitated the securing of research budget from other organizations (inside and outside the health system)?**

🗌 Yes 🗌 No

**11- Have the results of this research been utilized by you and/or other researchers to define the following projects? (this question does not include article citation)**

🗌 Yes Number …………..

🗌 No

🗌 I do not know

**Section E: Impact on Decision-making**

**12- Have the research results been utilized in systematic review studies?**

🗌 Yes 🗌No/I do not know

**13- Have the research results been utilized in the development of one of the documentations below?** (Please note that “is not relevant” means that your study cannot itself be utilized in documentation development. For example, basic science studies are not expected to be used in the development of educational content for patient and/or people. By “no” we mean that even if the possibility of utilization in the following documentations exist, they have not been utilized for these items.)

| **Row** | **Type of study** | If your answer is yes please write the name of the document |
| --- | --- | --- |
|  | Clinical guideline or public health guideline  🗌 Yes 🗌No 🗌Is not relevant |  |
|  | Health Technology Assessment  🗌 Yes 🗌 No 🗌 Is not relevant |  |
|  | Educational content for patient and/or the public (such as patient decision aid etc.)  🗌 Yes 🗌 No 🗌 Is not relevant |  |
|  | Policy brief (A policy brief is a document that is prepared to present the options of policymaking for decision-making which contains scientific evidence on the advantages and disadvantages of policy options)  🗌 Yes 🗌 No 🗌 Is not relevant |  |
|  | Policy bills, guidelines and/or executive organizations’ legislations  🗌 Yes 🗌 No 🗌 Is not relevant |  |

**14- Have the results of this research been utilized in the compilation of a book?**

🗌 Yes 🗌 No 🗌 Is not relevant

Please write the name of the book and its year of publication if your answer is yes:

…………………………………………………………………………………………………………………………………………

…………………………………………………………………………………………………………………………………………

**15- Have the research results been utilized in the development of educational content for professional groups (education and continuing education of students)?**

🗌 Yes 🗌 No 🗌 Is not relevant

Please write the name of the educational content if your answer is yes:

…………………………………………………………………………………………………………………………………………

**16- Has the research been conducted upon demand of a specific organization and/or policymakers, managers and/or the industry?**

🗌 Yes 🗌 No

Please write the name of the concerned organization if your answer is yes:

…………………………………………………………………………………………………………………………………………

**17- Have the research results been utilized in the Health Ministry’s policymaking (directly or indirectly)?**

🗌 Yes 🗌 No 🗌 Is not relevant

If your answer is yes, please explain how:

…………………………………………………………………………………………………………………………………………

…………………………………………………………………………………………………………………………………………

**18- Have the research results been utilized in policymaking outside the health system (directly or indirectly)?**

🗌 Yes 🗌 No 🗌 Is not relevant

If your answer is yes, please explain how:

…………………………………………………………………………………………………………………………………………

…………………………………………………………………………………………………………………………………………

**19- Have the research results been utilized in the policymaking of your local context?**

🗌 Yes 🗌 No 🗌 Is not relevant

If your answer is yes, please explain how:

…………………………………………………………………………………………………………………………………………

…………………………………………………………………………………………………………………………………………

**20- Have the research findings resulted in the registration of a domestic patent or invention?**

🗌 Yes 🗌 No 🗌 Is not relevant

**21- Have the research findings resulted in the registration of an international patent or invention?**

🗌 Yes 🗌 No 🗌 Is not relevant

**Section F: Health impact**

**22- Do you expect the application of your research results to improve the level of health directly (e.g., disease prevalence and incidence, quality of life and/or life expectancy)?**

🗌 Yes 🗌 No

| If your answer is no, please go to Q. 25 |
| --- |

22.1- If your answer to Q. 24 is yes, have the research results actually been put to practice for this purpose?

🗌 Yes 🗌 No 🗌 I do not know

22.2- If your answer to Q. 22.1 is yes, have the desired results been achieved?

🗌 Yes 🗌 No 🗌 I do not know

22.3- If your answer to Q. 22.2 is yes, among the scientific evidence that have affected the resultant decision-making, how much was the share of your research?

🗌 Less than 25% 🗌25 – 50% 🗌50 – 75% 🗌 More than 75%

**23- Do you expect the application of these results to improve the status of health determinants (e.g. modifiable risk factors, social and bioenvironmental determinants)?**

🗌 Yes 🗌 No

| If your answer is no, please go to Q. 26 |
| --- |

23.1- If your answer to Q. 23 is yes, have the research results actually been put to practice for this purpose?

🗌 Yes 🗌 No 🗌 I do not know

23.2- If your answer to Q. 23.1 is yes, have the desired results been achieved?

🗌 Yes 🗌 No 🗌 I do not know

23.3- If your answer to Q. 23.2 is yes, among the scientific evidence that have affected the resultant intervention, how much was the share of your research?

🗌 Less than 25% 🗌 25 – 50% 🗌 50 – 75% 🗌 More than 75%

**24- Do you expect the application of these results to improve the status of health services delivery (e.g. acceptability, accessibility, suitability, efficiency, efficacy and safety)?**

🗌 Yes 🗌 No

| If your answer is no, please go to Q. 27 |
| --- |

24.1- If your answer to Q. 24 is yes, have the research results actually been put to practice for this purpose?

🗌 Yes 🗌 No 🗌 I do not know

24.2- If your answer to Q. 24.1 is yes, have the desired results been achieved?

🗌 Yes 🗌 No 🗌 I do not know

24.3- If your answer to Q. 24.2 is yes, among the scientific evidence that have affected the resultant intervention, how much was the share of your research?

🗌 Less than 25% 🗌 25 – 50% 🗌 50 – 75% 🗌 More than 75%

**Section G: Economic impact**

**25- Do you expect the research findings to result in the production of new materials or consumer services?**

🗌 Yes 🗌 No

| If your answer is no, please go to Q. 28 |
| --- |

25.1- If your answer to Q. 25 is yes, have the research results actually been put to practice for this purpose?

🗌 Yes 🗌 No 🗌 I do not know

25.2- If your answer to Q. 25.1 is yes, have the desired results been achieved?

🗌 Yes 🗌 No 🗌 I do not know

25.3- If your answer to Q. 25.2 is yes, among the scientific evidence that have affected the resultant intervention, how much was the share of your research?

🗌 Less than 25% 🗌 25 – 50% 🗌 50 – 75% 🗌 More than 75%

**26- Do you expect the research findings to result in improvement of goods and/or already existing products (increased quality and/or reduction of costs)?**

🗌 Yes 🗌 No

| If your answer is no, please go to Q. 29 |
| --- |

26.1- If your answer to Q. 26 is yes, have the research results actually been put to practice for this purpose?

🗌 Yes 🗌 No 🗌 I do not know

26.2- If your answer to Q. 26.1 is yes, have the desired results been achieved?

🗌 Yes 🗌 No 🗌 I do not know

26.3- If your answer to Q. 26.2 is yes, among the scientific evidence that have affected the resultant intervention, how much was the share of your research?

🗌 Less than 25% 🗌 25 – 50% 🗌 50 – 75% 🗌 More than 75%

**27- Do you expect the research findings to result in knowledge-based entrepreneurship?**

🗌 Yes 🗌 No

| If your answer is no, please go to Q. 30 |
| --- |

27.1- If your answer to Q. 27 is yes, have the research results actually been put to practice for this purpose?

🗌 Yes 🗌 No 🗌 I do not know

27.2- If your answer to Q. 27.1 is yes, have the desired results been achieved?

🗌 Yes 🗌 No 🗌 I do not know

27.3- If your answer to Q. 27.2 is yes, among the scientific evidence that have affected the resultant intervention, how much was the share of your research?

🗌 Less than 25% 🗌 25 – 50% 🗌 50 – 75% 🗌 More than 75%

**28- Do you expect the direct application of research findings to result in reduction of workdays missed because of illness or disability’ (hospital or house rest)?**

🗌 Yes 🗌 No

| If your answer is no, please go to Q. 31 |
| --- |

28.1- If your answer to Q. 28 is yes, have the research results actually been put to practice for this purpose?

🗌 Yes 🗌 No 🗌 I do not know

28.2- If your answer to Q. 28.1 is yes, have the desired results been achieved?

🗌 Yes 🗌 No 🗌 I do not know

28.3- If your answer to Q. 28.2 is yes, among the scientific evidence that have affected the resultant intervention, how much was the share of your research?

🗌 Less than 25% 🗌 25 – 50% 🗌 50 – 75% 🗌 More than 75%

**29- Do you expect the application of research findings to result in reduction of patients’ direct costs?**

🗌 Yes 🗌 No

| If your answer is no, please go to Q. 32 |
| --- |

29.1- If your answer to Q. 29 is yes, have the research results actually been put to practice for this purpose?

🗌 Yes 🗌 No 🗌 I do not know

29.2- If your answer to Q. 29.1 is yes, have the desired results been achieved?

🗌 Yes 🗌 No 🗌 I do not know

29.3- If your answer to Q. 29.2 is yes, among the scientific evidence that have affected the resultant intervention, how much was the share of your research?

🗌 Less than 25% 🗌 25 – 50% 🗌 50 – 75% 🗌 More than 75%

**30- Do you expect the application of research findings to result in reduction of the health system’s direct costs?**

🗌 Yes 🗌 No

| If your answer is no, please go to Q. 33 |
| --- |

30.1- If your answer to Q. 30 is yes, have the research results actually been put to practice for this purpose?

🗌 Yes 🗌 No 🗌 I do not know

30.2- If your answer to Q. 30.1 is yes, have the desired results been achieved?

🗌 Yes 🗌 No 🗌 I do not know

30.3- If your answer to Q. 30.2 is yes, among the scientific evidence that have affected the resultant intervention, how much was the share of your research?

🗌 Less than 25% 🗌 25 – 50% 🗌 50 – 75% 🗌 More than 75%

| *Thank you for lending your valuable time to us and helping us ‘measure the impact of health research’.*  *We are deeply grateful.* |
| --- |
